# Imagery about suicide in depression—“Flash-forwards”?

**DOI:** 10.1016/j.jbtep.2007.10.004

**Published:** 2007-12

**Authors:** Emily. A. Holmes, Catherine Crane, Melanie J.V. Fennell, J. Mark G. Williams

**Affiliations:** Department of Psychiatry, University of Oxford, UK

**Keywords:** Mental imagery, Suicide, Intrusive memory, Flashback, Depression, Transdiagnostic, Intrusive image, Image rescripting

## Abstract

Suicide is a significant world health problem, with more deaths by suicide globally than by war. We need to better understand the cognitive processes underlying suicidal thinking for improved treatment development. Cognitive psychology indicates that mental imagery can be causal in determining future behavior, yet the occurrence of suicide-related imagery has not previously been investigated. Interviews with 15 depressed and formerly suicidal patients in remission found that all patients reported experiencing detailed mental imagery in addition to verbal thoughts when at their most despairing, for example images of making a future suicide attempt. A clinical measure of the severity of suicidal ideation was associated with both preoccupation with suicide-related imagery and perceived imagery realness. Echoing flashbacks in posttraumatic stress disorder, the current images appeared like “flash-forwards” to suicide. These results provide the first data to our knowledge on the existence of mental imagery in suicidality, opening a promising new avenue for research.

## 1 Introduction

Suicide is now among the three leading causes of death worldwide among those aged 15–44 years. Suicide attempts are up to 20 times more frequent than completed suicide (World Health Organization, n.d.). The scale of the problem is illustrated in that “worldwide more people kill themselves than die in all wars, terrorist acts and interpersonal violence combined” (International Association for Suicide Prevention, 2006). Effective treatments for suicidality are woefully lacking. Suicidal behavior occurs most frequently during a depressive episode (Mann, Waternaux, Haas, & Malone, 1999). Recommended evidence-based treatments for depression are psychological approaches such as cognitive behavior therapies, also known as CBT (e.g. Hollon et al., 2005). CBT is also highly promising for patients who have recently attempted suicide (Brown et al., 2005). However, few effective treatments exist specifically for those recurrently depressed patients who show suicidal features (e.g. suicidal thinking) and are known to be at increased risk of eventual suicide. To support innovative treatment development, we need to better understand the underlying cognitive processes (Harvey, Watkins, Mansell, & Shafran, 2004; Salkovskis, 2005).

Mental imagery has been shown to be causal in determining future behavior, for example imagining voting and actually doing so later (Libby, Shaeffer, Eibach, & Slemmer, 2007). Imagining an event not only increases the likelihood of engaging in what is imagined (e.g. signing up for cable TV; Gregory, Cialdini, & Carpenter, 1982 or doing exam revision; Pham & Taylor, 1999) but also makes it appear more probable (e.g. winning an election or donating blood; Carroll, 1978). Imagery is well known to enhance memory, and is also proposed to be important in driving personal goals (Conway, 2001; Conway, Meares, & Standart, 2004). Further, imagery is associated with strong emotion-imagining short emotion-laden scenarios has a more powerful impact on affect than verbal processing of the same material (Holmes & Mathews, 2005).

If the imagined event is useful (e.g. navigating to a desired destination), the above consequences of mental imagery (i.e. on action, perceived probability, memorability and emotion) have clear benefits. However, if the imagined event relates to suicidal behavior, these properties have very different implications. Translated into the domain of suicidality, the potential impact of mental imagery to increase the likelihood of action, increase perceived event probability, enhance memorability, goal-focus and intensify emotionality, would become particularly concerning if about suicide. Cognitive therapy has long held that cognitions can take the form of images as well as verbal thoughts (Beck, 1976). We know that intrusive, affect-laden imagery is key to posttraumatic stress disorder (Ehlers & Clark, 2000) and social phobia (Hirsch, Clark, Mathews, & Williams, 2003), where such imagery can feel very real, compelling and distressing to patients. We know patients with depression can experience negative intrusive imagery of past trauma (Kuyken & Brewin, 1994). However, to our knowledge, no previous work has examined whether suicidal patients report experiencing mental imagery related to suicide, such as planning a future suicide attempt.

Mental health clinicians routinely ask patients about “suicidal thoughts” during risk assessment, but tend to focus on content rather than form (e.g. does the patient have specific plans about ending their life? If so, how lethal is the plan?). Such questions may fail to elicit the presence of suicidal images. Whilst there has been some previous reference to imagery in suicidality, the issue has received scant attention. Chiles and Strosahl (2005, p. 79) suggest that patients may have difficulty fighting off detailed images of previous suicide attempts and describe a patient who said “I imagine I just end it all” but not how. Berk, Henriques, Warman, Brown, and Beck (2004) developed a CBT intervention for patients with previous suicide attempts, which included a component where patients were asked to imagine a scenario likely to evoke suicidal thoughts to mind and then imagine an adaptive response. However, the utility of this component has not been evaluated and we neither know whether patients had spontaneously occurring images related to suicide nor what they consisted of.

In summary, the findings from cognitive psychology discussed above inspired us to explore whether images also play a role in suicide attempts. Both the cognitive and clinical literature, to our knowledge, suggest that this may be a useful but under-researched topic. This study aimed to examine: (a) whether recovered patients with a history of suicidal depression report experiencing any suicide-related imagery during crisis (including of previous suicide attempts, future plans, or being dead) and (b) the nature and content of any imagery reported, including distress/comfort, preoccupation and realness. We sought to test whether a clinical measure of suicidal ideation severity was associated with either the time spent thinking about such images or perceived image realness. As it was the first study of this type, we focused on patients in recovery, recently treated to provide skills to deal with the distress evoked by discussing suicidal episodes in the interview.

## 2 Method

### 2.1 Participant recruitment

Patients with a history of depression and suicidality (ideation and/or behavior) who had recently completed treatment in randomized controlled trial were invited to participate. This treatment was a form of cognitive therapy recommended for relapse prevention in patients with recurrent depression (mindfulness-based cognitive therapy, MBCT; NICE, 2004; Williams, Duggan, Crane, & Fennell, 2006). All participants were in recovery at the time of participation (defined according to NIMH criteria; Frank et al., 1991; Keller, Shapiro, Lavori, & Wolfe, 1982). Self-reports of suicidal ideation were operationalized according to the Beck Scale for Suicide Ideation—Worst-Ever Version (BSSw). Participants with current psychosis, eating disorder, OCD, severe alcohol or substance misuse, ongoing habitual self-cutting or insufficient written or spoken English were excluded. Patients with bipolar disorder were included if they had been free from manic symptoms for 6 months.

### 2.2 Assessment

#### 2.2.1 Mini International Neuropsychiatric Interview (MINI; Sheehan et al., 1998)

MINI is a well-established, structured psychiatric interview used to assess current and past diagnostic status.

#### 2.2.2 Beck Depression Inventory (BDI-II; Beck, Steer, & Brown, 1996)

BDI-II is a well-established 21-item questionnaire measure assessing depressive symptomatology over the preceding 2 weeks.

#### 2.2.3 Beck Scale for Suicide Ideation—Worst-Ever Version (BSSw)

The BSSw uses the first 19 items of the Beck Scale for Suicide Ideation (BSS; Beck & Steer, 1993; Beck, Steer, & Ranieri, 1988; Williams, Barnhofer, Crane, & Beck, 2005), but is worded in the past tense and requires respondents to report on the time they felt at their most down and depressed about life. The first five items produce a screening score and the remainder are only completed if the participant reports that they had an active desire to kill themselves or intended to take a chance on life or death in a life-threatening situation. Beck, Brown, Steer, Dahlsgaard, and Grisham (1999) suggest that worst-point (rather than current) suicidality represents a significant predictor of an individual's future risk of death by suicide. The current minimal criteria for defining suicidality were a BSSw score indicating a “weak/no wish to live” in conjunction with a “weak/moderate or strong wish to die”.

#### 2.2.4 Suicidal cognitions interview

A structured interview was developed based on previous clinical mental imagery studies (Day, Holmes, & Hackmann, 2004; Hackmann, Clark, & McManus, 2000; Holmes, Grey, & Young, 2005). The terms “verbal thought” and “mental imagery” were explained using examples and feedback elicited to confirm comprehension. A checklist was used to assess content of cognitions when participants were at their most despairing. Participants were asked whether they had experienced any images or verbal thoughts for each of nine topics (see Table 1), and to describe examples to check comprehension. All participants were able to readily identify distinct images. Participants identified the *most significant image* they experienced when at their most despairing or suicidal, and described it in detail. They were asked for any associated affect or meaning. Pilot work indicated that suicide-related images may be comforting as well as distressing so both were explored. Participants rated image *distress* and *comfort* on nine-point scales (1=not at all to 9=extremely). Participants rated how *real* these images felt (when at their most despairing) and the amount of *time* they experienced images specifically related to suicide (to assess preoccupation). Ratings were made on nine-point scales (1=not at all to 9=as if it were reality/all the time). Interviews were conducted by the first two authors.

### 2.3 Procedure

At entry to treatment, participants completed the diagnostic interview (MINI), BSSw and BDI-II. Following treatment (delivered by J.M.G.W. and M.J.V.F.), patients completed the suicidal cognitions interview. Participants were thoroughly debriefed. A clinical psychologist (E.A.H.) discussed how participants might use skills acquired during treatment if distress re-occurred.

## 3 Results

### 3.1 Participant characteristics

Fifteen of the 18 invited individuals participated (nine women, six men). The mean age was 41.10 (S.D.=8.41) and mean of years in education was 15.33 (S.D.=2.89). All participants were Caucasian.

### 3.2 Diagnostic status

The median number of prior depressive episodes was 4 (*M*=5.54, S.D.=5.06, range 1–20); two individuals reported episodes too numerous to count. All patients were in recovery with a mean BDI-II score of 15.27 (S.D.=12.77). While four participants reported active suicidal ideation at entry to treatment, none did at the time of the interview. Participants reported the following axis I disorders: alcohol abuse (3), social phobia (3), generalized anxiety disorder (2) and agoraphobia without panic disorder (2). History of co-morbid lifetime disorders was as follows: alcohol dependence (3), substance dependence (2), substance induced psychosis (1), bipolar I disorder (1), manic episode (1), psychotic symptoms (1), panic disorder with agoraphobia (3), panic disorder without agoraphobia (1), agoraphobia (1) and dysthymia (1).

### 3.3 Treatment history

In addition to MBCT, participants had previously received the following treatment: antidepressant medication (7), lithium and an antipsychotic drug (1), inpatient treatment for depression or mania (2), inpatient detoxification (1), and counseling or psychotherapy (12).

### 3.4 Severity of suicidal ideation

All participants reported previous suicidal ideation. Severity of the worst episode was assessed using the BSSw. The median time since this period was 66 months (*M*=83.20, S.D.=64.45, range 10–240 months). The mean screening score (first five BSSw items, maximum=10) was 7.40 (S.D.=2.95). Thirteen participants completed the whole BSSw, i.e. reported an active desire to kill themselves. The mean total score was 22.34 (S.D.=6.59) of a maximum of 38—greater than the cut off of 16 used to identify high-risk patients who had 14 times the risk of committing suicide of those in the low-risk range (Beck et al., 1999). This is also higher than in a study (mean total score 13.3, S.D.=7.4) where patients were demonstrated to have cognitive vulnerability related to suicidality (Williams et al., 2005). Four participants had made prior suicide attempts. A further two reported previous habitual self-cutting. The remainder had experienced suicidal ideation in the absence of suicidal behavior (see Table 2).

### 3.5 Experience of suicide-related imagery/verbal thoughts during crisis

The following results refer to cognitions during participants’ most despairing or suicidal period. Table 1 shows participants’ endorsement of the nine checklist items for content, for both imagery and verbal thought separately. Imagery was reported in addition to verbal thoughts for all nine topics. For two categories, significantly more images than verbal thoughts were reported. Using a Wilcoxon signed ranked test, images of “what might happen if you died” (10/15 participants) were more frequent than verbal thoughts (4/15 participants), *Z*=2.45, *N*=15, *p*=.014. Second, images of “planning or preparing to make a (new) suicide attempt or harm yourself” (10/15 participants) were endorsed more frequently than verbal thoughts (6/15 participants), *Z*=2.00, *N*=15, *p*=.046. No other comparisons were significant, *Z*<1.13. Thus, it was observed that for categories related to the future (e.g. attempts, other people's reactions) imagery appeared more prevalent than verbal thoughts.

### 3.6 Description of most significant image

All patients reported experiencing intrusive, repetitive suicide-related images when at their most depressed and despairing. For a summary (following Morrison et al., 2002), including image descriptions, affect and meaning, see Table 2. Three examples follow.

*Example 1*: *Image of future suicidal action with behavioral consequence*. The patient reported repeatedly seeing themselves jumping from a specific cliff and deciding whether to leave a final message or phone call. They described “looking at my feet and grass on one side and sea and rocks on the other side … and I just thought I’d jump”, describing this as resulting in “blank and black and nothing … it was like the ultimate salvation, my own choice”. This patient had a history of absconding from hospital, and attempting to travel to the actual cliff with the intention of acting on the image as described. They reported this image occurred even when mildly depressed.

*Example 2*: *Sensory image of dead self and funeral*. This patient described “I could feel it, that cold damp feeling and being in a coffin”. They also reported visual images of the funeral with details of the specific church. They noted that they did not imagine how other people would feel as this “did not comfort” them. During the descriptions, they began “reliving” the imagery, saying “the images are coming into my head now, I can see myself in the coffin, I’m looking in”.

*Example 3*: *Comforting image of location providing opportunity for suicide*. The repetitive image was of having the opportunity (rather than the aftermath which they did not want to visualize) of deliberately crashing their car at a particular spot on their everyday route. “It's like um, the road kind of comes like here and then the trees stop, but they grow over the top, it was sort of like there was almost a tunnel the trees going over the tunnel. And I kind of felt like I was being drawn towards the tunnel all the time. Which just dropped down …”. The patient reported that the image came “quite often when I was struggling, it looked like quite a welcoming site really … quite comforting in a way … An opportunity to escape … I think it made me more relaxed actually”. This patient had made plans including arranging accident cover and life insurance policies.

### 3.7 Qualities of suicidal imagery

#### 3.7.1 Image distress and comfort

All participants rated their suicide-related image when experienced at times of crisis as distressing (7/15 rating them as moderately distressing or greater). However, 12/15 participants also rated them as comforting (with 10/15 rating them as moderately comforting or greater). Ratings of distress (*M*=5.13, S.D.=3.38) and comfort (*M*=5.00, S.D.=2.75) were similar.

#### 3.7.2 Preoccupation with suicide-related images and relation to severity of suicidal ideation

About 10/15 patients reported being preoccupied with suicide-related mental images more than half the time during the crisis (*M*=6.07, S.D.=2.80). Increased preoccupation with images related to suicide was associated with higher scores on BSSw screening items (*rs*=.56, *p*=.029, *N*=15).

#### 3.7.3 Perceived realness of suicidal images and relation to severity of suicidal ideation

About 10/15 participants reported experiencing their suicide-related images as feeling at least half-real (rather than just a mental event) (*M*=6.70, S.D.=2.94). Higher levels of image reality were associated with higher “worst-ever” suicidal ideation (BSSw screening items, *rs*=.54, *p*=.036, *N*=15).

## 4 Discussion

We aimed to explore: (a) whether recovered patients with a history of depression and suicidality reported experiencing any suicide-related imagery (as well as verbal thoughts) during crisis and (b) the nature and content of such imagery, and whether a clinical measure of suicidal ideation severity was associated with either the time spent thinking about such images or perceived image realness. Our results suggested that when suicidal, participants did indeed experience mental imagery in addition to verbal thoughts for all content categories covered. They were more likely to experience imagery than verbal cognitions for two categories (what might happen if they died and for suicidal plans); interestingly, both of which were future-oriented. While all images were rated as distressing, most participants reported that images were also comforting. High scores on the clinical worst-ever suicidal ideation measure (BSSw) were significantly associated with higher levels of image reality and increased preoccupation with suicide-related images. Collectively these findings indicate the importance of understanding imagery in suicidal patients, and that this previously neglected area is an exciting avenue for future research.

All participants reported experiencing suicide-related mental images during crisis, including acting out future suicidal plans or being dead. As we argued in Section 1, imagery can have implications for future behavior (cf. also prospective cognitions; Chasteen, Park, & Schwarz, 2001). We suggest that the term “flash-forwards” to suicide may be used to describe these suicide-related images. This term relates to “flashbacks” to past trauma—a hallmark of PTSD (Brewin & Holmes, 2003), but to thoughts of future suicide rather than to past trauma. Flashback memories are rich sensory-perceptual images (Conway, 2001; Ehlers, Hackmann, & Michael, 2004; Holmes et al., 2005), rather than verbal thoughts, and are affect-laden, accompanied by a sense of reality or “nowness”—as if the past trauma is really happening. The “flash-forward” images reported here were also described as possessing sensory qualities, of being real and compelling, and rich in detail, as illustrated in Table 2. Inspection of the content of the most significant images related to suicide described in Table 2, alongside the imagery interview, indicates that the majority of participants’ suicide-related images (13/15) were of the future rather than only of the past.

Clinical risk assessment and the identification of cognitive (and potentially modifiable) predictors for suicide are notoriously difficult. Our clinical measure of suicidal ideation (which does not refer to imagery) was associated with both image preoccupation and realness, though the large size of these correlations should be treated with caution given the sample size. The associations provide at least a good indication that asking about imagery during suicide risk assessment might be a useful inclusion, since this is not currently part of current clinical screening interviews. Interestingly, several participants commented that clinicians had never directly asked about suicidal imagery. To the non-clinician it may appear odd that patients do not spontaneously report suicide-related imagery. However, across psychological disorders (including PTSD and social phobia), patients frequently only report verbal cognitions if asked about “thoughts”. To elicit information about emotional and potentially shameful imagery (including about flashbacks), specific prompting is typically needed.

Focusing on imagery has informed the development of highly successful and improved cognitive-behavioral treatments for posttraumatic stress disorder (Ehlers & Clark, 2000) and social phobia (Hirsch et al., 2003). We propose that considering flash-forward suicidal imagery may also aid treatment innovation. Tackling imagery can require different cognitive therapy strategies than for verbal thoughts, proving new opportunities as in PTSD and social phobia. For example, cognitive therapy for PTSD includes “imaginal reliving” (Ehlers & Clark, 2000; NICE, 2005), where patients imagine their trauma in detail and add information to update traumatic meaning of specific hotspot images (e.g. “I’m about to be shot” to “I wasn’t shot”).

Our data indicate several imagery features that therapy could target: changing the flash-forward outcome to an alternative to suicide, reducing imagery realness and preoccupation, and reducing comfort. Akin to work in PTSD and borderline personality disorder (Arntz & Weertman, 1999; Giesen-Bloo et al., 2006), “imagery rescripting” might be used to produce an alternative flash-forward outcome, e.g. a flash-forward to overdose could be rescripted to an image of disposing of the tablets (cf. Berk et al., 2004; Brown et al., 2005). In the context of this Special Issue, it is indeed of particular interest to consider the potential of imagery rescripting techniques. Weertman and Arntz (2007) used imagery with rescripting to change the meaning of schematic representations of the past. The majority of the images reported in the current paper were not of the past per se (though some may have reflected past suicide attempts), but of images of harming/killing the self in the future. It is speculated that perhaps just as we can rescript imagery of past moments, we may learn to rescript future-oriented images. For example, Arntz et al. ask the patient to enter the image as a “bystander”, and the therapist can then interview the bystander about his/her feelings, thoughts and inclinations, and thus learn how best to rescript the image. Similar techniques could be applied to suicidal imagery to incorporate alternative meaning and outcomes necessary for (and informed by) the individual patient, e.g. outcomes of safety rather than harm, alternative sources of comfort and so on.

“Comfort” associated with imagery may be positively reinforcing (cf. craving imagery; Kavanagh, Andrade, & May, 2005) and imagery techniques may be useful to break this circle of reinforcement. In our data, imagery reality (“nowness”) and preoccupation were associated with higher scores on the BSSw, so addressing these features could be beneficial. Interestingly, the feature of “nowness” has been related to severity of depression in intrusions of past events in a student sample (Williams & Moulds, in press), and this remains to be tested for suicidal “flash-forwards”. Mindfulness techniques (Williams et al., 2006) may, for example, help patients relate to their images as “images” rather than as reality, allowing them to explore alternative strategies to either suppressing them or becoming attached to them.

This novel data is limited by a small sample, requires replication, and should be extended to non-treated sample with active suicidal ideation (if adequate clinical support can be ensured post-interview). Also, the data do not bear on whether suicide-related imagery is specific to depression as opposed to other disorders. Further imagery properties need to be explored, e.g. observer perspective (McIsaac & Eich, 2004), vividness (Baddeley & Andrade, 2000) and so forth. However, in the clinical context of individual case histories, the data are extremely compelling. We have argued that the presence of mental imagery (as compared to verbal cognition) about an event can increase emotion, perceived likelihood, goal focus and even action, and that in the context of suicide this may pose significant risk. As a cognitive process not yet directly tackled in the suicidality literature, our data suggest that more extensive consideration of mental imagery about suicide may deliver benefits in an area where treatment innovation is sorely needed.

## Figures and Tables

**Table 1:** The number of participants endorsing each item on the checklist used to assess content of imagery and/or verbal thoughts when at their most despairing or suicidal

Category on checklist	Imagery		Verbal thoughts	
	No	Yes	No	Yes
Of a time you tried to harm yourself in the past	9	6	11	4
Of yourself planning/preparing to harm yourself or make a future suicide attempt	5	10	9	6
Of what might happen to you if you died	5	10	11	4
Of what might happen to other people if you died	5	10	6	9
Of things you were escaping from	2	13	3	12
Of another (non-suicide related) distressing event that happened to you (e.g. a trauma)	3	12	3	12
That made you feel safe or better	8	7	7	8
That were fleeting/unclear	7	8	6	9
Any other type	9	6	8	7

**Table 2:** Suicide-related images reported by patients when at their most despairing or suicidal, affect and meaning associated with the image, and the nature of the previous suicidal ideation or attempt(s)

Gender	Content of image	Associated affect	Associated meaning	Previous suicidal plans and/or attempt(s)
Male	Jumping from a cliff	Not scared, wanting to do it	Ultimate salvation, best prospect, my own choice. Escape from illness and psychotic idea I had spend eternity in hell on earth	Escaped from hospital. Jumped into river, tried to jump in front of traffic, tired to reach cliffs to jump, attempted hanging
Female	At the road side looking at aftermath of a crash I had been killed in	Initially relief. Later guilt and anxiety	It is over, I do not have to worry anymore	Thoughts of overdose. Identified places to find information on medication to take, but had not looked
Male	Jumping in front of a train; emergency services at the scene; family at the funeral	Sad, tearful, fear, anger, comfort	I can escape my problems, get comfort	Thoughts of cycling into traffic or jumping off buildings. Sat on building ledge contemplating jumping. Attempted suffocation
Female	Damp feelings, being in a coffin at church funeral	Very frightened, tearful, sad, as if bereaved, unable to lift out of it	I cannot cope, floundering, not being helped, got to get help. Why am I having this (image)? Want to be dead, peace	One attempted strangulation. Two attempted overdoses
Female	Being on a platform, throwing myself in front of a train	Calm at time of despair	Testing myself, am I prepared to do this? Testing what I am actually feeling	Daily threats to jump in front of train (6 weeks) Thoughts, but no plans, of other methods, e.g. overdose
Male	Killing myself by slashing my wrists	Want to howl, revenge	Difficulty meeting children's needs and demands. Get away and not be there, get attention	Cutting wrists. Hospitalized on two occasions
Male	Scene of my brother's suicide including his trainers	Fear, anxiety, trepidation	I do not want to go there, I hope I do not get that bad I can do something like that	Four episodes of “really bad suicidal thoughts” no concrete plans
Female	My dead parents, flipping from one face to another	Very sad, helpless	I am all alone. I am feeling low—I want my parents back. Suicide as a “way to end the blackness”	More than 11 episodes of escalating suicidal ideation
Male	Deliberately crashing my car in a specific place	Comfort	Opportunity to escape	Between 5 and 10 periods of suicidal ideation and planning. Prominent thoughts of crashing car
Female	Going to sleep after an overdose, looking peaceful	Going peacefully. Frightened of not waking up again	Take the tablets—end it all, go to sleep and never wake up	Two previous overdoses
Female	Jumping out of window contrasted with another image of taking pills	Horror, sadness of putting myself through this, potential relief of not feeling pain	Which way would be the most effective, and cause least suffering on route? I thought I might act on them	One period of suicidal ideation considering where to jump from. Collected medication for overdose
Female	Being sucked and pulled towards death	Relief, feeling of escape. Overwhelming, fighting	What it would be like if the mental pain stopped	Planned to drink vodka and die outside in the cold. Self-harm via cutting
Male	Holding a gun to my head, pulling the trigger and immediately shutting down	Momentarily reduces despair	Desire to escape, oblivion	Carelessly injecting drugs risking overdose
Female	Image of myself, oscillating perspectives, thinking it would be better not to exist and stop fighting this	Absolutely terrible, totally drained, disempowered, abdicating responsibility	I was seeing it so must be true. This is who I am, a bad person. It would be easier if I were not here. No drive, even for a suicide attempt	Approximately five episodes thinking of overdose, no concrete plans
Female	Seeing myself from outside in bed, slitting wrists with a penknife	Being ashamed of myself. Life as painfully dull	Obstacles, somehow, an impossibility of going forward	Thoughts of slitting wrists periodically throughout 20's. Recent period when suicide seemed only solution, but no concrete plans
